# Kennedy F Shortridge PhD (April 6, 1941 to November 8, 2020): Obituary

**DOI:** 10.1111/irv.12849

**Published:** 2021-04-16

**Authors:** Robert G. Webster, Yi Guan, Malik Peiris

**Affiliations:** ^1^ Department of Infectious Diseases St Jude Children's Research Hospital Memphis TN USA; ^2^ School of Public Health The University of Hong Kong Hong Kong China

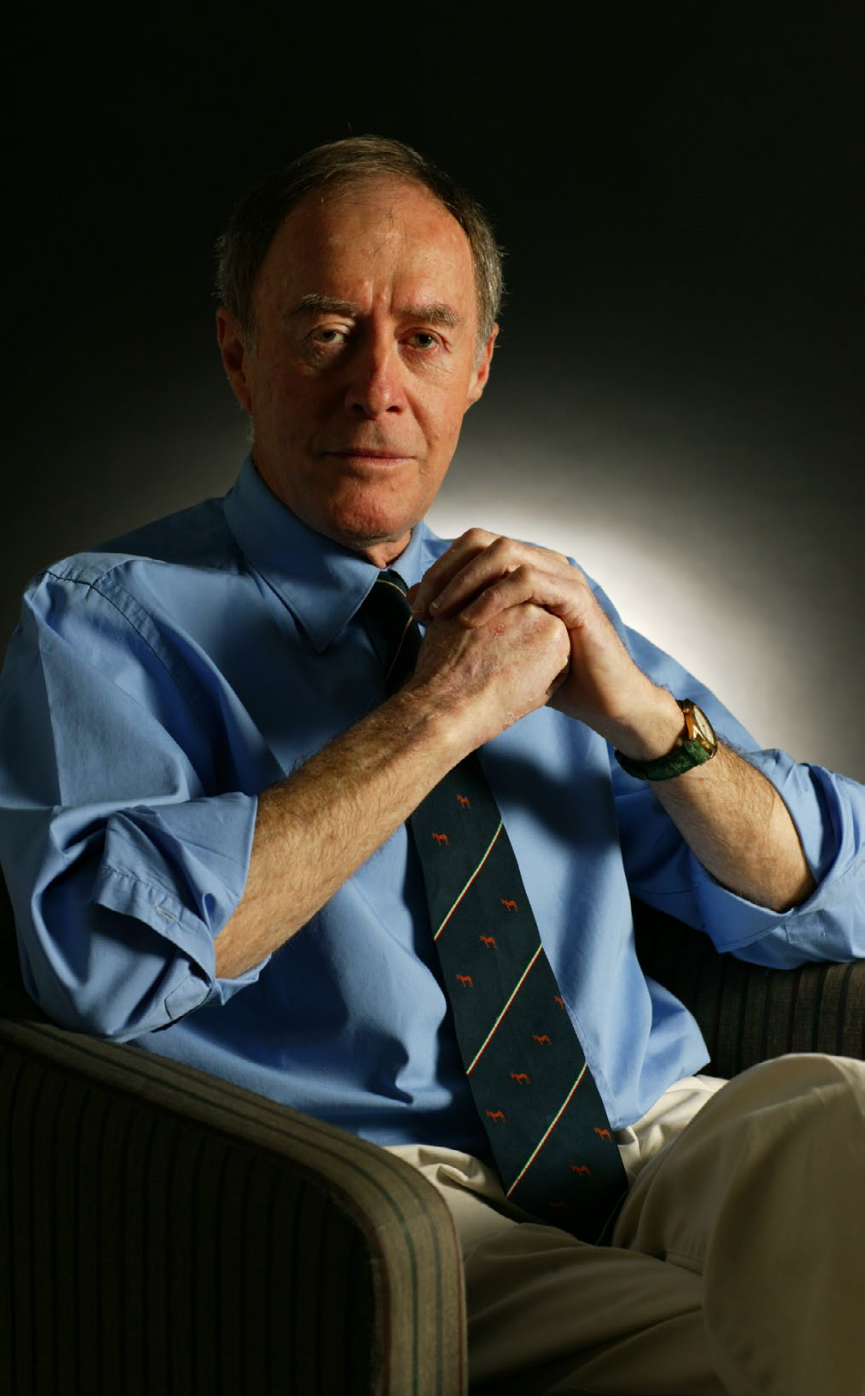

Scientific visionary, influenza ecologist, philanthropist, hill walker, rotarian, art collector, and sinophile are some of the descriptors for Kennedy F Shortridge (Ken). It is challenging to adequately detail the many contributions that Ken made to global public health and our knowledge of emerging viruses during his remarkable career at the University of Hong Kong.

Born an only child on the April 6, 1941, in Mount Isa, Queensland, Australia. At the age of 4, his family moved to Maryborough, Queensland, where he spent his early years. His father was a passionate bushwalker and a walking companion of pioneering conservationist Myles Dunphy, and his mother was a nurse who described vividly her childhood memories of the catastrophic 1918 Spanish influenza pandemic in the region. She described a man with a horse and cart who collected the many bodies during the pandemic. Ken won a scholarship to attend Nudgee College in Brisbane. He had a flair for running for which he earned several trophies. Later in life, he enjoyed hockey and squash.

After graduating from the University of Queensland with a BSc in microbiology, he moved to London, when he joined University College Hospital where human influenza virus was first isolated as a research assistant to Frank Biddle. Frank’s life was tragically cut short. Ken subsequently carried out research under the supervision of Prof G Belyavin on the structure of adenovirus type 5 at the University College Hospital Medical School, University of London and was awarded his PhD in 1971. Ken’s early research was on serum inhibitors of hemagglutination by several viruses including adenovirus, influenza and arboviruses which were potential confounders in viral serology assays.

While finalizing his PhD at University College Hospital, Ken met a visiting researcher from University of Hong Kong who recommended he apply for a position in a new department that was being set up there. In 1972, Ken joined the Department of Microbiology at the University of Hong Kong. Still moved by his mother’s stories about the 1918 pandemic, he decided to continue to focus his work on influenza. The primary focus of Ken’s research during his 30 years tenure in Hong Kong was the origin of pandemic influenza viruses from zoonotic reservoirs and the importance of collaboration with scientific colleagues in mainland China. Both the Asian H2N2 1957 and the Hong Kong H3N2 1968 influenza pandemics emerged in this region, so he was in an optimal position to initiate collaborative studies with Chinese virologists and with the influenza program of the World Health Organization (WHO). He initiated influenza virus surveillance in the live poultry markets and showed that most of the subtypes of influenza A viruses including the three subtypes (H1 H2 H3) that had caused pandemics in humans could be isolated from apparently healthy poultry. The highest frequency of isolation was from aquatic birds, principally from domestic and wild ducks and from chickens and quail. These studies culminated in his keynote paper with Sir Charles Stuart‐Harris in Lancet (1982) that southern China was an influenza epicenter, for ducks and pigs living near humans in the rice farming area created the optimal environment for interspecies transmission of influenza viruses. Validation of the concept was the emergence of the H5N1 bird flu virus in 1997 in apparently healthy animals in the live poultry markets in Hong Kong that spread to humans and caused up to 60% mortality. Closure of the live poultry markets in Hong Kong resulted in the initial cessation of spread of H5N1 to humans, but after reopening of the markets sporadic outbreaks in poultry and people occurred. This demonstrated that eventually live poultry markets should optimally be phased out. Ken became a chair professor in virology at the University of Hong Kong in 1981. In recognition of their contributions to the control of H5N1 influenza, Ken Shortridge and Margaret Chan were awarded the Prince Mahidol Award for service to the global community by the king of Thailand in 1999. Dr Chan subsequently served as the Director General of WHO from 2006‐2017.

The potential role of pigs in the genesis of novel influenza pandemics was the other arm of Ken Shortridge’s studies in Hong Kong. In collaboration with the World Health Organization, he organized surveillance studies for influenza in pigs in Wuhan, Chengdu, Guiyang, and Guangzhou provinces in mainland China as well as weekly surveillance in Hong Kong. He established that the H3N2 influenza viruses that had disappeared from humans were perpetuated in pigs. Additionally, he showed reassortment of avian influenza viruses and human influenza viruses in pigs. These studies also documented the introduction of avian influenza virus genes into pigs in China. Continuation of this program after Ken retired showed that swine influenza viruses from Europe and North America were moving across continents and helped explain the emergence of the 2009 H1N1 influenza pandemic from pigs in the Americas.

It was a real challenge to keep pace with Ken on an afternoon walk on the Dragon's Back hiking trail in Hong Kong. Ken was a keen walker, for this was one of his hobbies that he had learned from his father. Most people do not associate Hong Kong with mountain trails; there are many outstanding trails including Ma On Shan, Tai Mo Shan, with more gentle walks on Lantau and Lamma islands. One of the most outstanding is the gentle circular walking track around The Peak where on a clear day you can view the superb harbor and city. One of Ken’s optimal family vacations was to walk village to village in Italy, but his hobby of art collecting would sometimes delay the walking as he searched for local treasures to add to his collection.

Ken Shortridge was an active member of the international Rotary Club of Queensway in Hong Kong which he joined soon after its formation. He attended all meetings and brought new ideas in medical science to the attention of members. An important contribution to public health was Ken's recommendation for the club to support the vaccination of newborn babies in Hainan and Shandong provinces of China against Hepatitis B virus. From his frequent visits to these provinces, Ken learned that, despite being a free vaccine, the parents of newborn babies could not afford the fees charged for administration of the vaccine. Being a virologist Ken also knew that Hepatitis B was a serious problem in this region and that up to 90% of babies born to positive mothers would become chronic virus carriers. Later in life, they would develop severe liver disease with a high risk of developing liver cancer. Ken and his fellow Rotarians made numerous visits to these provinces to oversee the project. Their work was so successful that in 2000, it was elevated to being a major project supported by the Rotary clubs in the entire region. In collaboration with the Ministry of Health of China and a district‐wide donation of 20 million Hong Kong dollars, the program was expanded to 10 provinces of China. In recognition of their contribution to public health, Ken and the Queensway Rotarians were invited to China's Great Hall of the People in Beijing for formal recognition of their work in vaccinating the one millionth Chinese baby against hepatitis B. Ken's other contribution to the program was the donation of his $25,000 US prize money from the Prince Mahidol Award. He received the Paul Harris Fellows award from Rotary for his generosity and was expressly proud to contribute to this important project.

Ken was extremely generous in giving and sharing his ideas as a mentor of students and as an internationally respected scientist. He mentored a number of research students, some going on to achieve global recognition in their own right, including Dr Yi Pu Lin currently at the WHO Collaborating Centre for Reference and Research on Influenza, The Frances Crick Institute, London and Professor Yi Guan (one of the co‐authors) at the University of Hong Kong and Shantou University.

Ken was extremely knowledgeable about Chinese history and art, being able to point out the different styles of painting and pottery from the different dynasties in China. He enjoyed orchestral music, great food, and good wine. After retirement from the University of Hong Kong, he moved with his wife Joyce to Auckland, New Zealand, where he continued his association with Rotary, and spent many hours in the streets of the city raising funds for charity and washing dishes at a weekly lunch organized by the local church for new immigrants. The influenza scientists of the world are especially indebted to Ken for his role as a scientific conduit to China and for his work on influenza and other emerging zoonotic diseases. Ken is survived by his first wife Dilys, three adult children, four grandchildren, and by his second wife Joyce and her three children and three grandchildren.

## AUTHOR CONTRIBUTION

**Robert G Webster:** Conceptualization (equal); Writing‐original draft (lead); Writing‐review & editing (lead). **Malik JS Peiris:** Conceptualization (equal); Writing‐original draft (supporting); Writing‐review & editing (supporting). **Yi Guan:** Conceptualization (equal); Writing‐original draft (supporting); Writing‐review & editing (supporting).

### PEER REVIEW

The peer review history for this article is available at https://publons.com/publon/10.1111/irv.12849.

